# Comparison of postoperative clinical outcomes and knee stability of cruciate‐retaining total knee arthroplasty using the tibia‐first gap navigation technique with a computer‐aided system and measured‐resection technique: A retrospective analysis of a propensity‐matched cohort

**DOI:** 10.1002/jeo2.12084

**Published:** 2024-07-05

**Authors:** Masaki Iguchi, Tsuneari Takahashi, Ryusuke Ae, Katsushi Takeshita

**Affiliations:** ^1^ Department of Orthopedic Surgery Miyazaki Prefectural Nobeoka Hospital Nobeoka Japan; ^2^ Department of Orthopedic Surgery Jichi Medical University Shimotsuke Japan; ^3^ Department of Orthopedic Surgery Ishibashi General Hospital Shimotsuke Japan; ^4^ Division of Public Health, Center for Community Medicine Jichi Medical University Shimotsuke Japan

**Keywords:** anterior and posterior knee stability, computer‐assist surgery, tibia‐first technique, total knee osteoarthritis

## Abstract

**Purpose:**

This study aimed to clarify whether the range of motion (ROM), anterior and posterior (AP) stability and other clinical measures changed in patients who underwent tibia‐first total knee arthroplasty (TF‐TKA) using navigation with a computer‐aided system after surgery.

**Methods:**

This is a retrospective study and we conducted a matched cohort analysis of 60 measured resection (MR)‐TKAs and 52 TF‐TKAs performed by a single surgeon. All the surgeries used the same implant and approach. Baseline differences between the groups were adjusted using propensity score matching. We compared each patient's measured ROM and Oxford Knee Score (OKS) and performed knee AP laxity measurements by using a device during routine follow‐ups.

**Results:**

A total of 40 MR‐TKAs with a mean age of 73.5 ± 5.6 years and sex (male 10, female 30) were compared to 40 TF‐TKAs with a mean age of 74.0 ± 5.7 years and sex (male 13, female 27) at 2‐year follow‐ups. Two years postoperatively, there was a significant difference in the AP laxity at 30° of knee flexion between both groups (7.0 ± 3.4 mm vs. 5.2 ± 2.3 mm, *p* < 0.01). In contrast, no differences were found between both groups for knee flexion (120.8 ± 9° vs. 116.7 ± 9.8°, *p* = 0.07) and OKS score (41.8 ± 6.9 vs. 41.0 ± 5.9, *p* = 0.61).

**Conclusion:**

The AP stability in the midflexion obtained using the tibia‐first technique remained consistent even after 2 years. However, OKS and ROM were not significantly different from those of the MR‐TKA group.

**Level of Evidence:**

Retrospective comparative LEVEL III study.

**Abbreviations**
AP: anterior and posterior
BMI: body mass index
CAS: computer‐assisted surgery
CR: cruciate retaining
HKA: hip‐knee‐angle
MR: measured resection
MR‐TKA: measured‐resection total knee arthroplasty
OKS: Oxford Knee Score
ROM: range of motion
TEA: trans epicondylar axis
TF‐TKA: tibia‐first total knee arthroplasty
TKA: total knee arthroplasty

## BACKGROUND

The use of computer‐assisted surgery (CAS) for total knee arthroplasty (TKA) has become widespread since the mid‐2000s; it helps in the improvement of alignment in the sagittal and coronal planes and reduces the need for revision [7]. CAS also significantly improves the accuracy relative to the mechanical axis, reduces soft tissue release, decreases bone cut and may improve gap balance [8]. Recently, a novel CAS that can evaluate ligamentous status through the entire range of motion (ROM) has emerged. It contributes to the evaluation of gap balance not only at 0° or 90° but also at any other knee position, including the intermediate flexion position. However, several authors have reported an inability to consistently and accurately position the femoral component in terms of internal/external rotation in the axial plane, even with the use of CAS [2, 14].

Tibia‐first total knee arthroplasty (TF‐TKA) uses the tibial resection plane to guide femoral component rotation, which eliminates the need to determine individual landmarks on the distal femur to identify a reference axis and avoids releasing soft tissue, thereby preserving its integrity. This may have the advantage of predicting the final soft tissue balance before femoral osteotomy [9]. As soft tissue balance affects femoral rotation, the tibia‐first technique is a method that can be used to improve femoral component rotational alignment. In addition, correct femoral rotation obtained using the navigated Whiteside's line and trans epicondylar axis (TEA) was inferior to the femoral rotation achieved using the tibia‐first technique [7].

Lee et al. reported that 94.4% of knees undergoing TKA achieved accurate femoral component rotations (within ±5°) using the tibia‐first technique [7]. In a previous study, Takahashi et al. reported that this technique has the potential to provide high postoperative anterior and posterior (AP) stability in the intermediate flexion position compared to the measured‐resection (MR)‐TKA [16], and intraoperative 30° AP stability was a positive predictive factor for obtaining satisfactory knee flexion [15].

To our knowledge, few reports have assessed the results of TKA using the tibia‐first technique with the novel CAS, and mid‐ to long‐term outcomes have not yet been reported. This study aimed to verify the hypothesis that the knee flexion and mid‐range AP stability would be superior 2 years postoperatively in TKA performed using both the novel CAS and tibia‐first technique.

## METHODS

### Ethics approval

This study was conducted in accordance with the principles of the Declaration of Helsinki. Our Institute's Bioethics Committee for Medical Research approved the study and waived the requirement for informed consent from individual participants due to the study's retrospective nature and all patients received standard treatment (approval ID: A20‐067).

### Participants

We included 112 patients who underwent primary TKA for knee osteoarthritis or varus knee deformities between April 2018 and March 2021. Sixty patients underwent MR‐TKA and the other 52 underwent TF‐TKA. The patients were divided into two groups: MR‐TKA and TF‐TKA.

We excluded patients with previous instances of TKA, knee osteotomy, previous collateral ligament injury or anterior or posterior cruciate ligament reconstruction because of the confounding effects of prior surgery on postoperative ROM. Patient age, body mass index (BMI), hip‐knee‐angle (HKA; varus indicates plus), ROM for both extension and flexion and Oxford Knee Score (OKS) were recorded. The ROM was measured using a double‐armed goniometer. OKS was defined on a scale of 0 (*worst*) to 48 (*best*). We excluded 20 patients due to lack of data and those who could not follow‐up 2 years after surgery. Ultimately, 92 patients were included in this study.

To adjust for baseline differences between the groups, a propensity score algorithm was used to match the MR‐TKA group with and TF‐TKA groups in a 1:1 ratio. The patients were matched for age, sex, BMI, HKA and pre‐ROM. Propensity score matching was performed between the 40 patients in the MR‐TKA group and 40 patients in the TF‐TKA group. After matching, age, sex, BMI, HKA, pre‐and postoperative ROM for extension and flexion and postoperative OKS were compared. In addition, stability at 30° and 90° of knee flexion was measured during a routine follow‐up visit using a Kneelax 3 arthrometer, which can calculate AP laxity by performing the AP drawer test to apply a force of 132 N to the tibia relative to the femur anteriorly and posteriorly, as described in a previous study by Takahashi et al. [16].

### Surgical procedure

All TKAs were performed using the midvastus approach, a cemented, fixed‐bearing prosthesis, pneumatic tourniquets, and the same cruciate‐retaining (CR) TKA implant (ATTUNE, DePuy Synthes). The anterior cruciate ligament was dissected and the posterior cruciate ligament was retained in each group. None of the patients underwent patellar resurfacing. Mechanically aligned TKA was performed to achieve neutral coronal mechanical limb alignment by cutting the femoral and tibial bones such that the rectangular flexion and extension gaps were perpendicular to the mechanical axes. All surgeries were performed by a senior board‐certified orthopaedic surgeon.

MR‐TKA was performed using a conventional technique, according to the manufacturer's instructions. First, a distal femoral cut was created using an intramedullary alignment system. Posterior femoral cuts were made with an anterior reference guide to make the bony resection line parallel to the surgical epicondylar line and perpendicular to Whiteside's line. A proximal tibial cut was made using an extramedullary alignment system. The collateral and retinacular ligaments were not released to balance the flexion and extension gaps.

**Figure 1:**
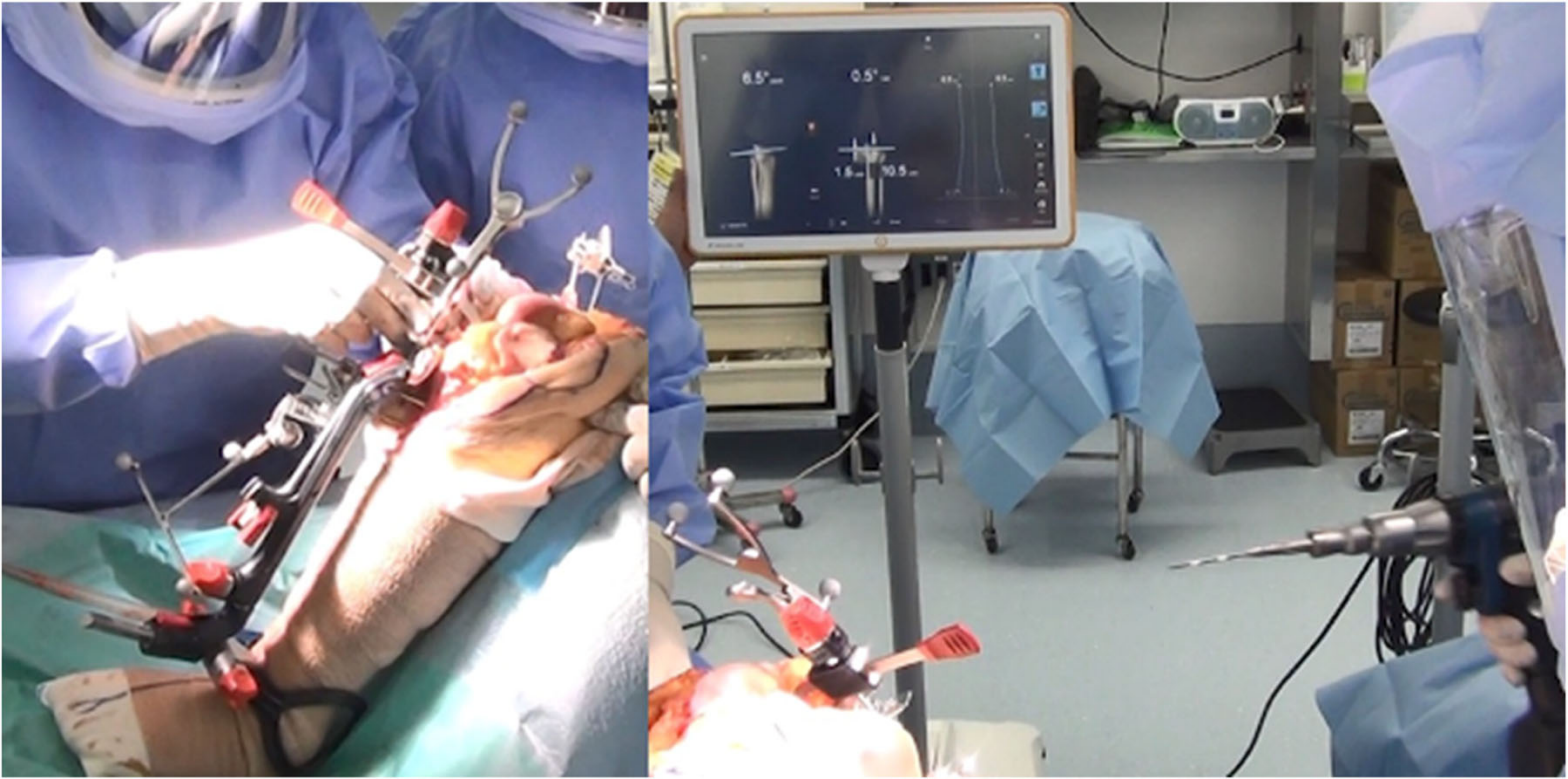
Bone resection was performed using an extramedullary bone cutting guide. By combining the manual guides with navigation, we installed a bone‐cutting guide while confirming the amount of bone resection as well as varus/valgus and posterior tilt.

**Figure 2:**
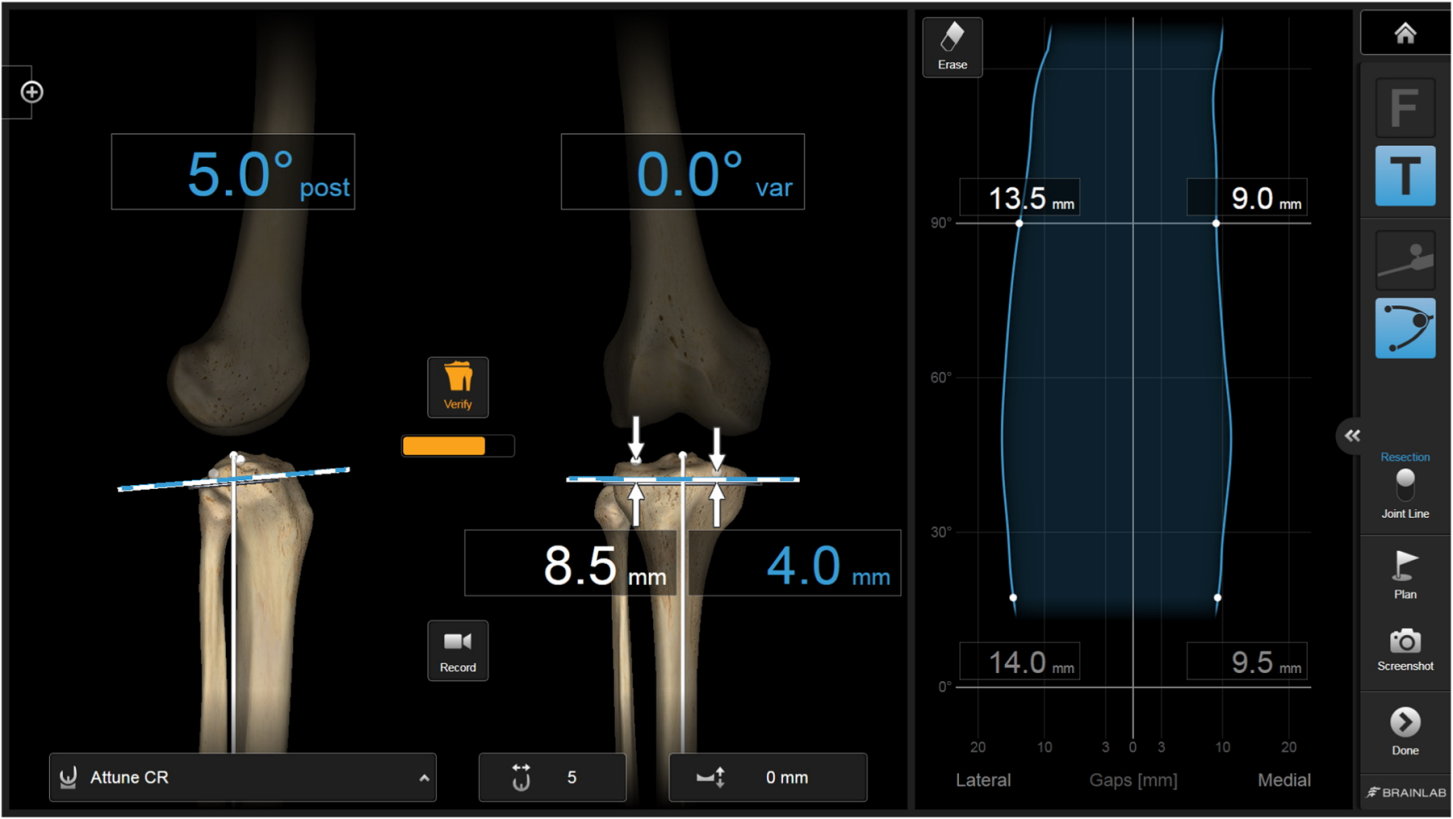
(CAS screen display) The varus/valgus angle, posterior tilt and assumed bone resection amount were displayed for the anticipated bone cuts. On the left screen, the vertical and horizontal axes represent the knee angle and the gap, respectively. Medial and lateral gaps throughout the entire ROM of the knee joint are indicated. CAS, computer‐assisted surgery; ROM, range of motion.

In the tibia‐first technique, tibial osteotomy is first performed to ensure that tibial bony resection is perpendicular to the anatomical tibial axis. Anatomical landmarks and individual biomechanical knee data were obtained using CAS (Kick; Brainlab AG) (Figures 1 and 2). Following the tibial bony resection, the medial and lateral gap sizes were measured dynamically using a tibial trial component. No release of medial collateral ligament was observed. The extent of distal and posterior femoral osteotomies and varus‐valgus balance were determined and the femoral component position was planned by navigation. In addition, we confirmed that appropriate identical medial and lateral gaps were observed throughout the ROM.

### Clinical outcomes and AP laxity 2 years after surgery (primary outcome)

The AP laxity measurements obtained using the Kneelax were determined as the primary outcome, and postoperative clinical evaluations included ROM for both extension and flexion. Two years after surgery, each patient underwent knee AP laxity measurements using Kneelax 3 during a routine follow‐up visit, as described in a previous study by Shi et al. [13, 16]. The same knee surgeon performed the AP drawer test in the same manner as the intraoperative measurement. OKS was also recorded during a routine follow‐up visit.

### Statistical analysis

Numerical variables were normally distributed. The groups were propensity score‐matched to minimise bias. Specifically, we used single‐nearest‐neighbour matching (also known as one‐to‐one matching). In this study, every tibia‐first case was matched with a measured resection case based on age, sex, BMI, HKA and pre‐ROM using propensity score matching, ensuring that the characteristics of the observed patients closely mirrored each other [6]. Outcomes of interest were compared using *X* ^2^ test (or Fischer's exact test) and Student's *t* test for categorical and continuous variables, respectively.

A prior sample size calculation using the unpaired *t* test for the primary outcome was performed, and the significance level was set at *p* < 0.05. The minimum sample size for an α error of 0.05, *β* error of 0.20 and effect size of 0.8 was 52 patients (using G Power 3.1, Franz Paul) [4]. In this study, we adopted the propensity score matching method, with each group comprising 40 patients. Therefore, based on post‐hoc power analysis with a sample size of 40, assuming an alpha error of 0.05ss and an effect size of 0.8, the power was 94.2%. All statistical analyses were performed using EZR software [5].

## RESULTS

### Comparisons of patient backgrounds

Eighty patients (40 in the TF‐TKA group and 40 in the MR‐TKA group) were included. No significant differences in male‐to‐female ratio, mean age, preoperative HKA or pre‐OKS were observed between the groups (Table 1).

**Table 1:** Comparison of the patient characteristics in the MR‐TKA and TF‐TKA groups.

	MR‐TKA	TF‐TKA	*p* Value
Age	73.5 ± 5.6	74.0 ± 5.7	0.74
Male/female	10/30	13/27	0.62
Pre HKA	12.3 ± 7.2 varus	10.7 ± 5.5 varus	0.29
Pre extension	−9.0 ± 5.8	−7.6 ± 7.8	0.37
Pre flexion	119.5 ± 11.5	124.3 ± 11.5	0.069
Pre OKS	23.4 ± 8.4	23.6 ± 8.9	0.90

*Note*: Data are expressed as the mean ± standard deviation.

Abbreviations: HKA, hip‐knee angle; MR‐TKA, measured resection‐total knee arthroplasty; OKS, Oxford Knee Score; TF‐TKA, tibia‐first total knee arthroplasty.

### Comparisons of the bony resection width

No significant differences were observed in the width of the bony resection in the distal femur and proximal tibia on the medial and lateral sides. In contrast, the width of bony resection of the posterior condyle on the medial and lateral sides was significantly smaller in the TF‐TKA group than in the MR‐TKA group (medial: 8.5 ± 2.0 mm vs. 10.2 ± 1.4 mm, lateral: 6.4 ± 1.6 mm vs. 7.1 ± 1.0 mm) (Table 2).

**Table 2:** Comparisons of the width of the bony resection measurements.

	MR‐TKA	TF‐TKA	*p* Value
Distal femur (mm)			
Medial	7.0 ± 1.4	7.5 ± 1.3	0.11
Lateral	6.9 ± 1.2	7.0 ± 1.4	0.90
Posterior femur (mm)			
Medial	10.2 ± 1.4	8.5 ± 2.0	<0.01*
Lateral	7.1 ± 1.0	6.4 ± 1.6	0.01*
Proximal tibia (mm)			
Medial	3.6 ± 2.2	4.4 ± 1.9	0.060
Lateral	10.8 ± 1.8	10.7 ± 1.4	0.81

*Note*: Data are expressed as the mean ± standard deviation.

Abbreviations: MR‐TKA, measured resection‐total knee arthroplasty; TF‐TKA, tibia‐first total knee arthroplasty.

* *p* < 0.05.

### Comparisons of the postoperative AP laxity and postoperative clinical outcomes 2 years after surgery

**Figure 3:**
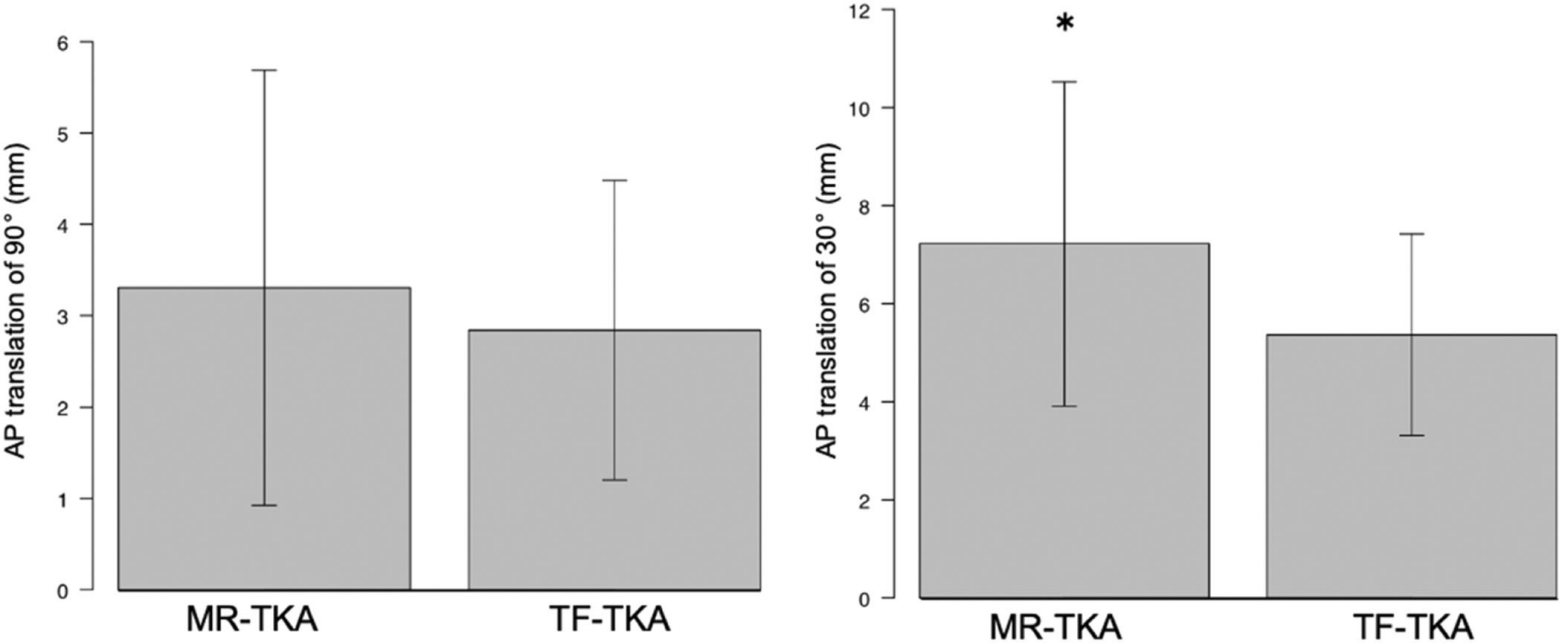
Knee AP laxity measurements obtained using Kneelax 3 arthrometer after 2 years after surgery. Translation was significantly smaller in the TF‐TKA group than that in the MR‐TKA group for AP laxity with 30° of knee flexion (5.2 ± 2.3 mm vs. 7.0 ± 3.4 mm). On the other hand, no significant AP laxity was observed for 90° knee flexion (2.7 ± 1.7 mm vs. 3.1 ± 2.2 mm). AP, anterior and posterior; MR‐TKA, measured resection‐total knee arthroplasty; TF‐TKA, tibia‐first total knee arthroplasty.

Translation was significantly smaller in the TF‐TKA group than that in the MR‐TKA group for AP laxity with 30° of knee flexion (5.2 ± 2.3 mm vs. 7.0 ± 3.4 mm). On the other hand, no significant AP laxity was observed for 90° knee flexion (2.7 ± 1.7 mm vs. 3.1 ± 2.2 mm) (Figure 3). Additionally, there were no significant differences between the two groups after 2 years of OKS and knee flexion (120.8 ± 9° vs. 116.7 ± 9.8°, *p* = 0.07) (Table 3).

**Table 3:** Comparisons of the 2‐year postoperative measurements.

	MR‐TKA	TF‐TKA	*p* Value
Knee flexion	120.8 ± 9.9	116.7 ± 9.8	0.069
Post 2 years OKS	41.8 ± 6.9	41.0 ± 5.9	0.61
Knee laxity measurements			
AP translation of 30°(mm)	7.0 ± 3.4	5.2 ± 2.3	<0.01*
AP translation of 90°(mm)	3.1 ± 2.2	2.7 ± 1.7	0.36

*Note*: Data are expressed as the mean ± standard deviation.

Abbreviations: AP, anterior and posterior; MR‐TKA, measured resection‐total knee arthroplasty; OKS, Oxford Knee Score; TF‐TKA, tibia‐first total knee arthroplasty.

* *p* < 0.05.

## DISCUSSION

This study has several findings. First, mid‐range AP stability was smaller 2 years postoperatively in TF‐TKA performed using the novel CAS than in MR‐TKA. Second, there were no significant differences in the OKS or ROM.

To the best of our knowledge, there have been no reports on propensity score‐matching outcomes 2 years after CR and TF‐TKA. Mid‐range AP stability 2 years after surgery was lower in the TF group than in the MR group. Takahashi et al. reported that the mid‐range of AP stability after surgery, assessed under anaesthesia, was smaller in the TF group than in the MR group [16], which was in accordance with the results of this study. It has been suggested that TF‐TKA with CAS may provide long‐term stability in the mid‐range position. It has commonly been observed that soft tissue release is significantly reduced by computer navigation [8]. Matziolis et al. reported that the reverse gap technique with navigation reduces soft tissue release and achieves mid‐range flexion stability [10]. Additionally, the tibia‐first technique has been reported to prevent a mismatch between the rotation of the femoral component and osteotomy of the posterior condyle [9]. We believe that navigation allows us to maintain the joint line even with the gap technique and install the CR‐TKA. In the tibia‐first technique, osteotomy of the posterior femur is performed after the tibia is cut, enabling osteotomy of the posterior femur while confirming the gap tension. Matziolis et al. reported that a posterior condylar offset of >2 mm caused mid‐flexion instability [11]. In this study, the width of the bony resection on the dorsal side was significantly smaller in the TF‐TKA group than that in the MR‐TKA group. We speculate that this may have conferred stability in the midflexion position. The implant elements might have also played a role in the mid‐range flexural stability. The implants used in this study were gradually reducing radius design implant. Clary et al. showed that among different femurs modelled with a gradually reducing radius design, an attenuating effect on the paradoxical anterior femoral slide was observed in traditional dual‐radius implants in their cadaveric study [3]. This gradually reducing radius design, combined with posterior condylar osteotomy and decreased rotation mismatch of the femoral component might have improved the mid‐flexion stability.

However, there were no differences in the ROM or OKS between TF‐TKA and MR‐TKA. Becker et al. reported that there was no significant difference between TF‐TKA and MR‐TKA using computer‐assisted surgery in the joint space and Knee Society Score, Short Form‐36 and knee injury and osteoarthritis outcome score [1]. Takahashi et al. reported that mid‐range AP stability was a positive predictive factor for achieving satisfactory knee flexion [15], which was not in line with the results of this study. Minoda et al. reported that a good preoperative flexion angle is a significant predictor of a good postoperative flexion angle after posterior‐stabilised TKA [12], and the relevance of other factors to ROM needs to be considered.

TF‐TKA with CAS could possibly improve mid‐range stability, thereby enhancing patient satisfaction. There was no difference between OKS and flexion ROM in this study. More cases are needed to investigate whether AP stability affects long‐term results and the effect of TF on patient satisfaction and ROM.

We did not assess the interobserver variation in the instrumental measurements. However, Kneelax 3 is an aximetry measurement and force control. All TKA surgeries were CR‐TKAs performed by a single consultant knee surgeon who used the mid‐vastus approach at a single facility. Therefore, these results cannot be generalised to TKAs using other approaches, such as minimally invasive and standard median parapatellar approaches, as well as posterior stabilised‐ or cruciate‐substituting TKA. Although propensity matching scores were used for assessment, this was not a randomised controlled trial and a potential bias might have existed. This is a limitation of this study. However, this is a topic for future investigation.

## CONCLUSION

Midrange AP stability 2 years postoperatively was superior in the group with TF‐TKA performed using the novel CAS compared to the MR‐TKA group. However, OKS and ROM did not differ significantly between the two groups.

## AUTHOR CONTRIBUTIONS

The author(s) read and approved the final manuscript.

## CONFLICT OF INTEREST STATEMENT

The authors declare no conflict of interest.

## ETHICS STATEMENT

This study was conducted in accordance with the principles of the Declaration of Helsinki. Our Institute's Bioethics Committee for Medical Research approved the study and waived the requirement for informed consent from individual participants due to the study's retrospective nature and all patients received standard treatment (Approval ID: A20‐067).

## Data Availability

The data that support the findings of this study are openly available.
